# Emphysematous Gastritis Secondary to Embolic Infarction: A Case Report

**DOI:** 10.7759/cureus.97407

**Published:** 2025-11-21

**Authors:** Yat Cheung Chung, Niyaz Naqash

**Affiliations:** 1 General Surgery, Monash Health, Melbourne, AUS; 2 Upper Gastrointestinal and Hepatobiliary Surgery, Monash Health, Melbourne, AUS

**Keywords:** embolic infarct, gastrectomy, gastric necrosis, splenic infarcts, surgical laparotomy

## Abstract

We report the case of a 73-year-old female with diet-controlled diabetes mellitus and atrial fibrillation on apixaban who presented with acute postprandial epigastric pain, nausea, and vomiting. The CT imaging revealed portal venous gas, intramural gastric gas, and splenic infarction, raising concern for emphysematous gastritis with associated ischemia. She underwent urgent total gastrectomy with Roux-en-Y reconstruction, which demonstrated full-thickness gastric necrosis and splenic infarction. Postoperatively, she recovered uneventfully following multidisciplinary management, including broad-spectrum antibiotics, nutritional support, and transition from apixaban to warfarin.

This case illustrates the diagnostic challenges of emphysematous gastritis, which may present with subtle clinical findings despite catastrophic pathology. The presence of portal venous gas and associated infarction should prompt urgent surgical evaluation. Early recognition and timely intervention can improve outcomes in this otherwise highly fatal condition.

## Introduction

To our knowledge, the last reported case of embolic infarct of the stomach was published in 1972 [1]. The first reported case by Baumann was an autopsy in 1909. A subsequent case series published in 1951 by Cohen further identified four more patients on autopsy showing gastric infarction secondary to an embolic event [2]. The last published case by Harvey et al. in 1972 identified and operated on a patient with what appeared to be a gastric ulcer at the time, but unfortunately also passed away, only to identify gastric infarction secondary to embolic infarct on autopsy. Embolic infarct of the stomach is an unusually rare pathology owing to its rich collateral blood supply, making this a rare cause of emphysematous gastritis.

Emphysematous gastritis is a rare but life-threatening infection of the stomach wall caused by gas-forming organisms. It is characterized radiologically by the presence of intramural gas and may be associated with portal venous gas, which carries a high mortality rate of up to 62%. Differentiating emphysematous gastritis from the more benign gastric emphysema is essential, as the latter is usually related to iatrogenic or mechanical injury and does not typically require surgical intervention [3,4].

Reported risk factors for emphysematous gastritis include diabetes mellitus, alcohol abuse, immunosuppression, and gastric malignancy. Clinical presentation is often non-specific, ranging from abdominal pain and nausea to features of sepsis, making early diagnosis challenging. While some patients may be managed conservatively with broad-spectrum antibiotics and supportive care, surgical intervention is indicated in cases with transmural necrosis, peritonitis, or associated infarction [5-8].

We present a case of emphysematous gastritis with concurrent splenic infarction in a patient on therapeutic anticoagulation with apixaban. This case underscores the diagnostic challenges, the potential for embolic etiology despite anticoagulation, and the importance of timely surgical management to achieve favorable outcomes.

## Case presentation

A 73-year-old female presented with an acute onset of epigastric pain after a steak meal. It was associated with nausea and vomiting without fever or bowel changes. She denied any other risk factors for peptic ulcer disease. She has a past medical history of diet-controlled type 2 diabetes mellitus, atrial fibrillation, which has been managed with apixaban and metoprolol, carotid endarterectomy, chronic kidney disease, left subclavian artery stenosis, and gastroesophageal reflux disease.

The patient last had her apixaban the evening before the presentation. She was hemodynamically stable on presentation, with a blood pressure of 191/57 mmHg and a heart rate of 79 beats per minute, and was afebrile. Her abdomen was soft with generalized tenderness without obvious distension or peritonism. Her white cell count (WCC) was 17.7 x10^9/L (reference range: 4.0-11.0x10^9/L), C-reactive protein (CRP) was 4.3 mg/L, hemoglobin (Hb) was 127, international normalized ratio (INR) was 1.3, estimated glomerular filtration rate (eGFR) was 39 ml/min (patient’s baseline), and apixaban level was 213 ng/ml, which was within the expected range for a 5 mg dose at peak level.

**Figure 1:**
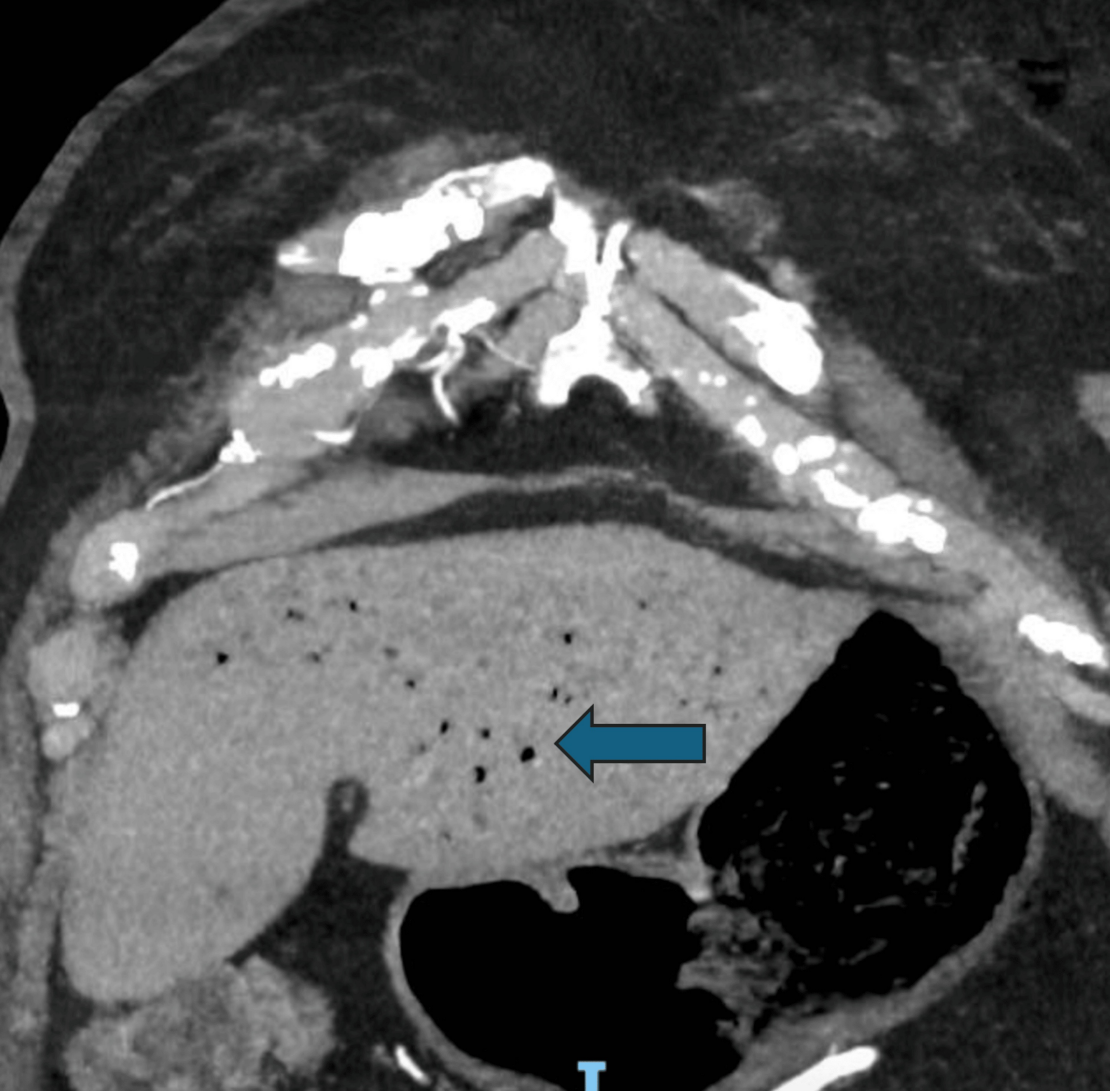
CT angiography showing portal venous gas (arrow)

**Figure 2:**
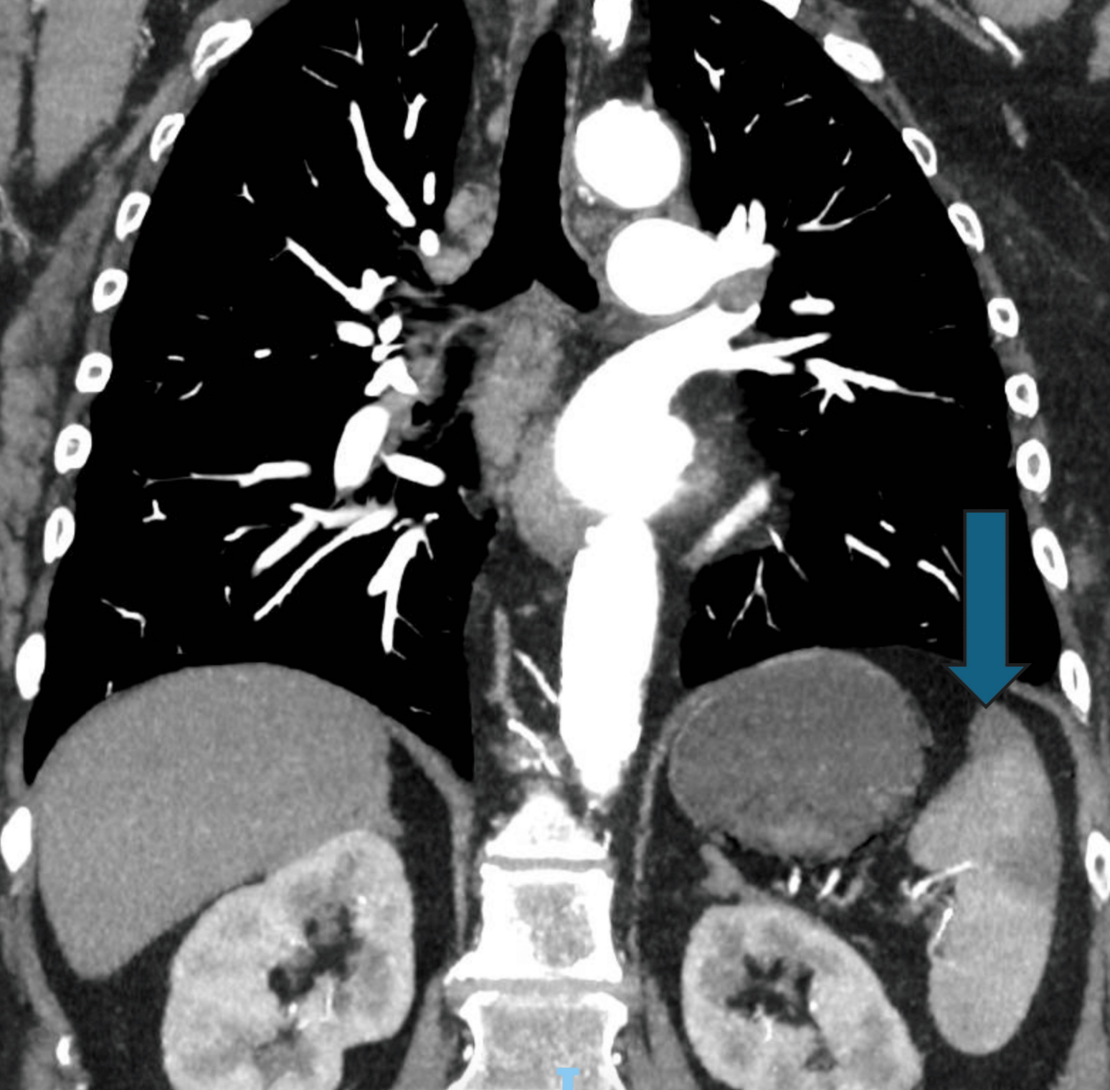
CT angiography showing the splenic infarct (arrow)

**Figure 3:**
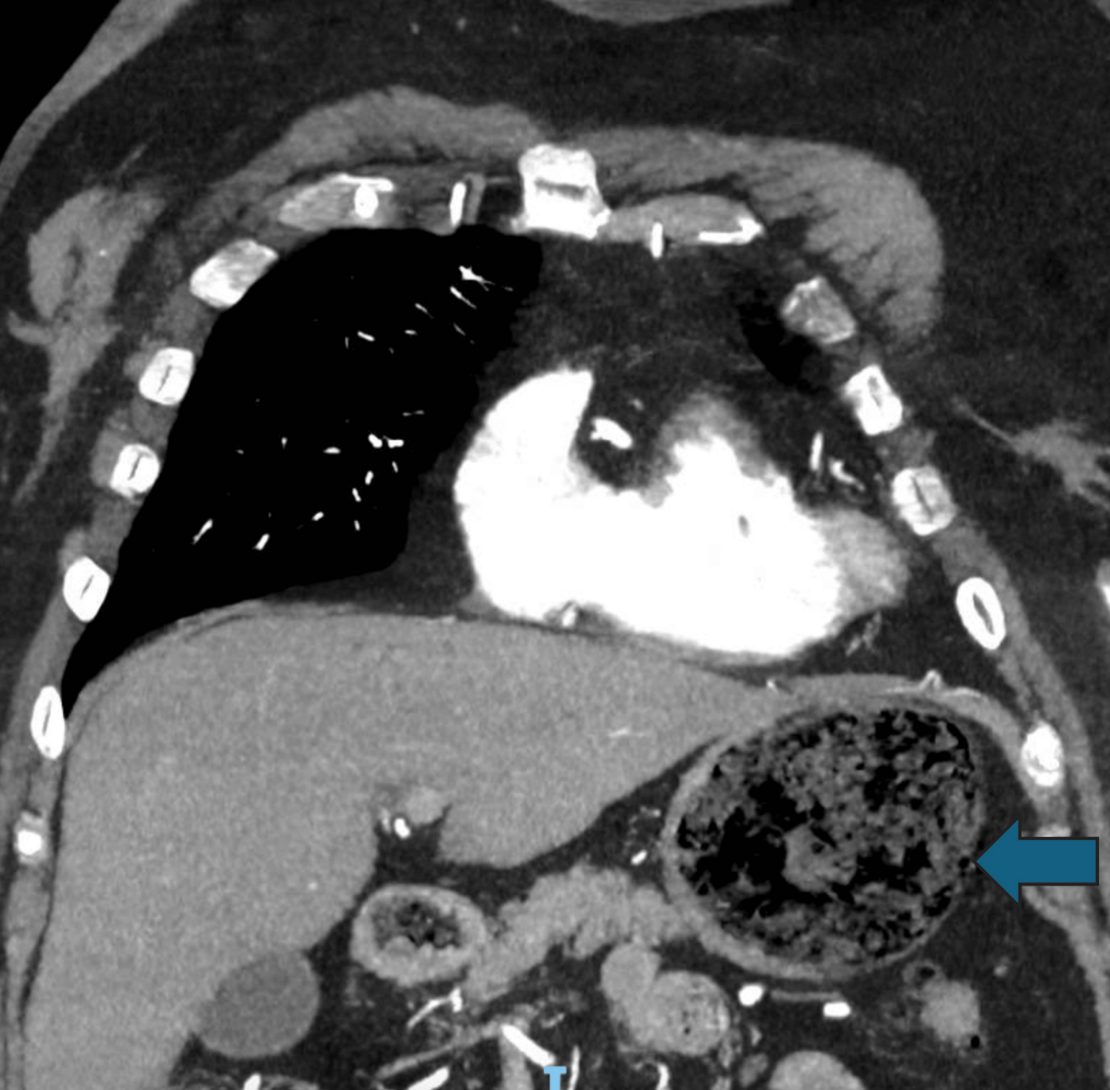
CT angiography showing mild intramural gas within the stomach (arrow), suggestive of emphysematous gastritis

Whilst the patient was in the emergency department, an urgent CT scan with intravenous contrast of the thorax was organized to rule out aortic dissection. The CT scan instead revealed portal venous gas (Figure 1), wedge-shaped perfusion defects of the spleen (Figure 2), and intramural gas in the stomach (Figure 3). This was suggestive of potentially multiple embolic incidents resulting in emphysematous gastritis and splenic infarct. No obvious arterial occlusions of the celiac branches were identified on the CT scan otherwise. The patient was started on broad-spectrum antibiotics and was resuscitated. 

**Figure 4:**
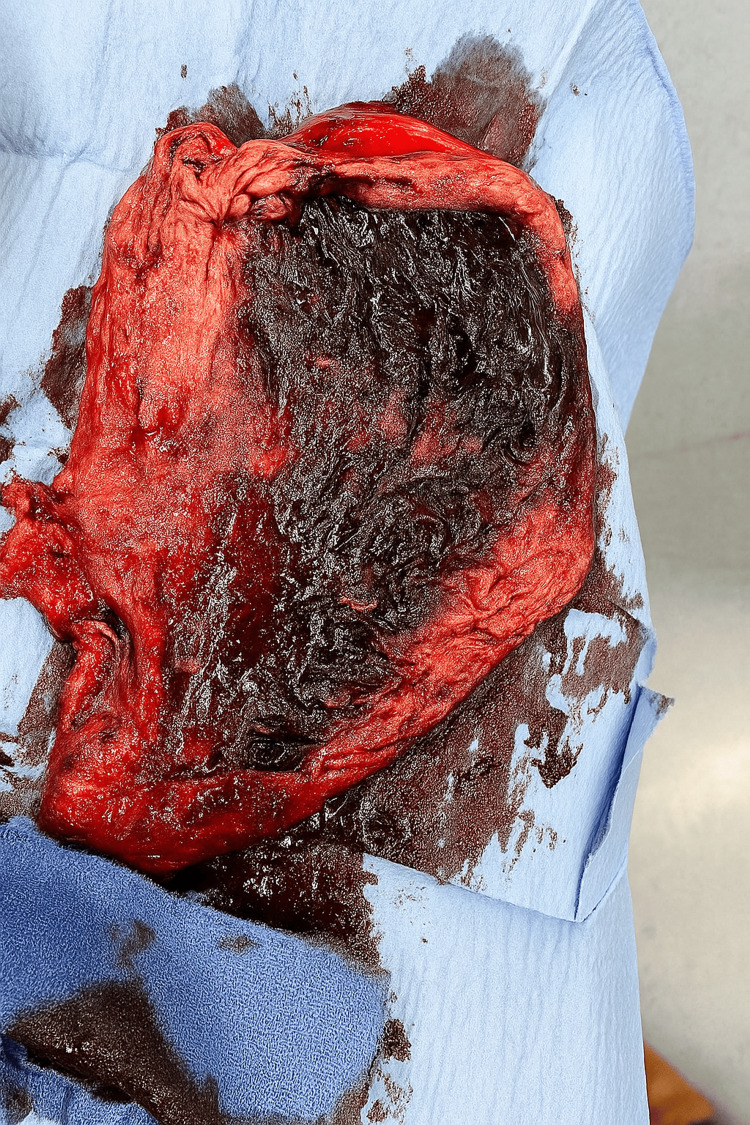
Intraoperative total gastrectomy specimen showing large area necrosis in the posterior stomach

An urgent gastroscopy, open total gastrectomy, and Roux-en-Y bypass surgery were performed. On gastroscopy, there were no obvious malignant lesions or gastric ulcers. Intraoperative findings included full-thickness necrosis of the gastric fundus along the greater curvature and the posterior stomach, turbid fluid in the lesser sac, and an infarct involving 1/3 of the spleen (Figure 4). There was also no evidence of serosal or peritoneal nodules. This raised clinical suspicion for spontaneous infarction of the posterior stomach and spleen, likely secondary to embolic infarction despite anti-coagulation with apixaban. 

The patient was transferred to the high dependency unit (HDU) for postoperative care. She was kept on intravenous antibiotics for five days total per the infectious disease team’s guidance. A gastrograffin follow-through study performed five days postoperatively showed no anastomotic leak, and total parenteral nutrition (TPN) was ceased after nine days as guided by the clinical nutrition medical unit. The hematology team was consulted regarding the spontaneous gastric necrosis secondary to embolism. The patient’s apixaban was ceased and changed to warfarin given her total gastrectomy, nutritional status, and spontaneous clots. The patient overall had an uneventful recovery and was discharged on day 13 after the operation. 

The patient’s histopathology showed a broad area of gastric necrosis with complete and near-complete mucosal necrosis with hemorrhage within the lamina propria. It did not demonstrate a thrombus within the submucosal or subserosal vessels. The patient’s tissue and fluid microscopy, culture, and sensitivities did not grow any organisms. The operative findings and absence of organisms raise a high suspicion for gastric necrosis secondary to embolic infarct.

## Discussion

Emphysematous gastritis is a rare but life-threatening form of gastritis, with a mortality rate as high as 62% [3,4]. It is essential to distinguish between emphysematous gastritis and gastric emphysema. The former results from infection of the gastric wall by gas-forming organisms, most commonly *Escherichia coli*, *Streptococcus*, *Enterobacter clostridium*, and *Pseudomonas aeruginosa*, and carries a grave prognosis. In contrast, gastric emphysema is typically a benign finding, usually secondary to mucosal injury, upper gastrointestinal tract lesions, or iatrogenic injuries, such as nasogastric tube insertion or barotrauma during gastroscopy [5-8].

Patients with emphysematous gastritis are often also found to have risk factors such as alcoholism, immunosuppression, diabetes, ingestion of non-steroidal anti-inflammatory agents, or gastric tumors [9,10]. In this case, our patient only had diet-controlled diabetes as a risk factor. As the disease progresses, patients can become septic with worsening abdominal pain, nausea, and vomiting. In our case, the patient presented hemodynamically stable with vague postprandial epigastric pain, allowing for a long list of differential diagnoses. The CT is often the next mode of investigation. In the setting of emphysematous gastritis, the presence of portal venous gas indicates a higher severity of disease and a higher mortality rate [11,12].

Previous case reports suggest that emphysematous gastritis in the absence of full-thickness gastric ischemia can be managed conservatively with broad-spectrum antibiotics, fluid resuscitation, and observation [13-15]. However, additional CT findings of portal venous gas should prompt urgent surgical interventions, including gastroscopy, laparoscopy, or laparotomy, and total or partial gastrectomy, as patients are at risk of transmural ischemia and peritonitis [16].

Literature studies suggest the most common causes for emphysematous gastritis include malignant gastric tumors, small bowel obstruction, and acute dilation of the stomach. In our case, the findings of a wedged splenic infarct and partial gastric necrosis suggest a thrombotic or embolic incident that resulted in spontaneous gastric necrosis, as well as splenic infarct. Based on the operative findings, our patient would be classified as a late presentation of gastric necrosis; however, the signs and symptoms of the patient were vague and mild. Thus, highlighting the diagnostic challenges that may result in surgeons delaying the diagnosis and management of a surgical catastrophe. 

## Conclusions

This case highlights a rare presentation of emphysematous gastritis complicated by gastric and splenic infarction, likely secondary to an embolic event despite anticoagulation with apixaban. The patient’s vague initial symptoms and hemodynamic stability underscored the diagnostic challenge, as CT findings ultimately guided urgent surgical intervention. While emphysematous gastritis is often associated with high mortality, timely recognition, prompt surgical management, and multidisciplinary postoperative care led to a successful outcome in this patient. Clinicians should maintain a high index of suspicion in patients presenting with portal venous gas and intramural gastric gas, even in the absence of classic risk factors, as early intervention can be lifesaving.
